# Omega-3 supplementation during unilateral resistance exercise training in older women: A within subject and double-blind placebo-controlled trial

**DOI:** 10.1016/j.clnesp.2021.09.729

**Published:** 2021-12

**Authors:** M.S. Brook, Usu Din, J. Tarum, A. Selby, J. Quinlan, J.J. Bass, N. Gharahdaghi, C. Boereboom, H. Abdulla, M.V. Franchi, M.V. Narici, B.E. Phillips, J.W. Williams, F. Kadi, D.J. Wilkinson, P.J. Atherton, K. Smith

**Affiliations:** a MRC-Versus Arthritis Centre for Musculoskeletal Ageing Research and NIHR Nottingham BRC, Clinical, Metabolic and Molecular Physiology, School of Medicine, University of Nottingham, Derby, UK; b School of Life Sciences, University of Nottingham, Nottingham, UK; c School of Health Sciences, Örebro University, Örebro, Sweden; d School of Sport, Exercise and Rehabilitation Sciences, University of Birmingham, UK; e National Institute for Health Research, Birmingham Biomedical Research Centre at University Hospitals Birmingham NHS Foundation Trust, Birmingham, UK; f Department of Biomedical Sciences, University of Padova, Padova, Italy

**Keywords:** Omega 3, Skeletal muscle, Aging, Women, Protein synthesis

## Abstract

**Background & aims:**

The skeletal muscle anabolic effects of n-3 polyunsaturated fatty acids (n-3 PUFA) appear favoured towards women; a property that could be exploited in older women who typically exhibit poor muscle growth responses to resistance exercise training (RET). Here we sought to generate novel insights into the efficacy and mechanisms of n-3 PUFA alongside short-term RET in older women.

**Methods:**

We recruited 16 healthy older women (Placebo n = 8 (PLA): 67±1y, n-3 PUFA n = 8: 64±1y) to a randomised double-blind placebo-controlled trial (n-3 PUFA; 3680 mg/day versus PLA) of 6 weeks fully-supervised progressive *unilateral* RET (i.e. 6 × 8 reps, 75% 1-RM, 3/wk^−1^). Strength was assessed by knee extensor 1-RM and isokinetic dynamometry ∼ every 10 d. Thigh fat free mass (TFFM) was measured by DXA at 0/3/6 weeks. Bilateral vastus lateralis (VL) biopsies at 0/2/4/6 weeks with deuterium oxide (D_2_O) dosing were used to determine MPS responses for 0–2 and 4–6 weeks. Further, fibre cross sectional area (CSA), myonuclei number and satellite cell (SC) number were assessed, alongside muscle anabolic/catabolic signalling via immunoblotting.

**Results:**

RET increased 1-RM equally in the trained leg of both groups (+23 ± 5% n-3 PUFA vs. +25 ± 5% PLA (both P < 0.01)) with no significant increase in maximum voluntary contraction (MVC) (+10 ± 6% n-3 PUFA vs. +13 ± 5% PLA). Only the n-3 PUFA group increased TFFM (3774 ± 158 g to 3961 ± 151 g n-3 PUFA (P < 0.05) vs. 3406 ± 201 g to 3561 ± 170 PLA) and type II fibre CSA (3097 ± 339 μm^2^ to 4329 ± 264 μm^2^ n-3 PUFA (P < 0.05) vs. 2520 ± 316 μm^2^ to 3467 ± 303 μm^2^ in PL) with RET. Myonuclei number increased equally in n-3 PUFA and PLA in both type I and type II fibres, with no change in SC number. N-3 PUFA had no added benefit on muscle protein synthesis (MPS), however, during weeks 4–6 of RET, absolute synthesis rates (ASR) displayed a trend to increase with n-3 PUFA only (5.6 ± 0.3 g d^−1^ to 7.1 ± 0.5 g d^−1^ n-3 PUFA (P = 0.09) vs. 5.5 ± 0.5 g d^−1^ to 6.5 ± 0.5 g d^−1^ PLA). Further, the n-3 PUFA group displayed greater 4EBP1 activation after acute RE at 6 weeks.

**Conclusion:**

n3-PUFA enhanced RET gains in muscle mass through type II fibre hypertrophy, with data suggesting a role for MPS rather than via SC recruitment. As such, the present study adds to a literature base illustrating the apparent enhancement of muscle hypertrophy with RET in older women fed adjuvant n3-PUFA.

## Abbreviations

n-3 PUFA: n-3 polyunsaturated fatty acids
EPA: eicosapentaenoic acid
DHA: Docosahexaenoic acid
D_2_O: Deuterium oxide
MVC: Maximal voluntary contraction
RET: Resistance exercise training
1-RM: One repetition maximum
TC/EA: High temperature conversion elemental analyser
GC: Gas chromatography
IRMS: Isotope ratio mass spectrometer
MPS: Muscle protein synthesis
ASR: Absolute synthesis rate
DXA: Dual-energy X-ray absorptiometry
VL: Vastus lateralis
MCME: N –Methoxycarbonyl methyl ester
APE: Atom percent excess
RPE: Rating of perceived exertion
PLA: Placebo
TTM: Thigh fat free mass
CSA: cross sectional area
SC: satellite cell

## 1 Introduction

Age-related declines in skeletal muscle mass and function (sarcopenia) remains a significant healthcare problem, and finding means by which to mitigate sarcopenia continues to be an area of great interest and scientific endeavour. In the absence of effective and safe pharmacological strategies, much work continues to focus upon muscle loading paradigms and nutritional interventions aimed at effectively promoting muscle anabolism. In particular, resistance exercise training (RET) can increase both muscle mass and function in older age [1] – albeit to a marked lesser extent than in younger cohorts (especially regarding muscle hypertrophy [[2], [3], [4]]). This phenomenon is particularly true in older women, who exhibit negligible hypertrophic capacity [5,6]. In the absence of supportive nutrition, RET remains a catabolic stimulus [7], with the intake of sufficient dietary protein necessary to provide the essential amino acid (EAA) substrate to facilitate muscle anabolism (i.e. hypertrophy) over prolonged periods of RET. This is exemplified by acute protein turnover studies demonstrating that intake of dietary proteins (or free EAA) augment the anabolic response above exercise alone, while reciprocally acting to limit muscle proteolysis (presumably due to the anti-catabolic effects of insulin) [8,9]. While sufficient dietary protein is required for muscle building, the efficacy of numerous other nutritionally based compounds have undergone investigation as ergogenic aids when adjuvant to RET. Such nutrient compounds include, but are not limited to, Vitamin D [10,11], anti-oxidant vitamins (i.e. Vitamin C & E) [12], leucine metabolites [13] and compounds with anti-inflammatory properties (i.e. ibuprofen) [14].

Fish-oil derived compounds (principally docosahexaenoic acid (DHA) and eicosapentaenoic acid (EPA)) have been the subject of a significant body of pre-clinical and clinical work in the setting of general health promoting effects (e.g. reducing risks of heart disease [15], as anti-hypertensives [16], in reducing cholesterol [17], and in reducing joint pain [18]). Dietary sources of DHA and EPA include fatty fish, such as salmon, mackerel, trout, and shellfish. There have also been a number of studies into their anabolic effects in relation to skeletal muscle in catabolic diseases such as cancer [19], sarcopenia [20] and as a pro-anabolic supplement adjuvant to exercise [21]. Nonetheless, research outcomes have been mixed. Whilst some studies have shown no benefit of n-3 polyunsaturated fatty acids (PUFA) on acute anabolism or hypertrophic adaptations [22,23], it has been reported that 6-months of n-3 PUFA supplements can increase muscle mass and function, even in the absence of RET [20,24], while also augmenting muscle anabolism under hyperinsulinaemic and hyperaminoacidaemic conditions [25,26]. Similarly, a study in 2012 [21] and a more recent clinical trial [22], demonstrated that long term n-3 PUFA supplements, alongside RET, were effective in augmenting muscle quality and function, especially in older women (i.e. for the latter study in both sexes, however the positive effects in men were limited). This finding of a lack of effect in older men was also supported in a more recent placebo-controlled trial (RET for 3 months) - however only men were studied [27]. Dietary analysis of older men and women revealed that with each additional portion of fatty fish consumed per week resulted in a greater increase in grip strength in women [28]. The potential gender benefits of n-3 PUFA consumption has been suggested to arise from greater changes in n-3 PUFA plasma and membrane ratios [29,30]. Yet older men and women receiving 3g of n-3 PUFA/d showed no sex differences in erythrocyte EPA and DHA incorporation, whilst gender differences in muscle lipid uptake were not reported [22]. As such, the precise biological mechanisms that underlie possible sex effects of n-3 PUFA supplementation on muscle mass adaptations remain unclear.

Given the apparent leaning towards positive effects of n-3 PUFA for the maintenance of muscle mass and quality in older women in particular, we sought to generate insights into the efficacy and mechanisms of n-3 PUFA supplements adjuvant to RET in older women. We focused on a shorter-term trial than previously attempted, as arguably it provides a more practicable, deliverable intervention, and due to the fact that adaptation to RET has been shown to occur during the early phase of RET [31,32]. It was hypothesised that the provision of n-3 PUFA would augment RET adaptations by cumulative increases in MPS mediated via increased translational efficiency.

## 2 Materials and methods

### 2.1 Ethics and subject characteristics

This study was approved by The University of Nottingham Faculty of Medicine and Health Sciences Research Ethics Committee, complied with the Declaration of Helsinki (October 2013) and was registered at https://clinicaltrials.gov/(NCT02505438). Sixteen healthy older females were recruited and randomly assigned (8 per group) to either placebo (PLA) or n-3 PUFA supplementation with baseline characteristics described in Table 1. Sample size was powered based on the primary end point of chronic MPS. A sample size of 8 in each group was determined to have >80% power to detect a mean difference in MPS of 25% assuming that the pooled SD of chronic MPS measures is 0.23 (based on previous data from our lab [33,34]), and a Cohen's d estimation of effect size of 1.55 using a two-group unpaired t-test with a 0.05 two-sided significance. All power calculations were performed using the pwr R package (https://www.r-project.org). Subject inclusion criteria were all older (65-75y) women who are generally healthy and recreationally active. Subject exclusion criteria were, active cardiovascular disease, cerebrovascular disease including previous stroke, aneurysm (large vessel or intracranial), respiratory disease including pulmonary hypertension or COPD, hyper/hypo parathyroidism, hyper/hypothyroidism, cushing's disease, diabetes, active inflammatory bowel disease, renal disease, malignancy, recent steroid treatment (within 6 months), or hormone replacement therapy, clotting dysfunction, musculoskeletal or neurological disorders and any disease requiring long-term drug prescriptions including statins. All subjects were screened by medical questionnaire, physical examination, and resting electrocardiogram. Subjects had normal blood chemistry, were normotensive and were not prescribed any medications; all subjects performed activities of daily living and recreation but did not undertake any formal exercise regime. All subjects provided their written, informed consent to participate after all procedures and risks (in relation to muscle biopsies, blood sampling, etc.) were explained. All analysts and those involved in the conduct of the study were blinded throughout as to which treatment group the samples or subjects belonged.

**Table 1:** Baseline Characteristics. Data as mean ± SEM.

	Placebo	Supplement
**Age (y)**	66.5 ± 1.4	64.4 ± 0.8
**Height (m)**	1.58 ± 0.02	1.62 ± 0.02
**Weight (kg)**	64.3 ± 1.9	70.5 ± 2.6
**BMI (kg.m**^**−2**^**)**	25.8 ± 0.9	26.0 ± 0.7
**ASM (kg)**	15.4 ± 0.8	17.1 ± 0.5
**ASMI (ASMkg.m**^**−2**^**)**	5.9 ± 0.3	6.1 ± 0.2
**Activity Counts**	46,972 ± 10,481	57,749 ± 9189

### 2.2 Study conduct

Following inclusion, subjects were studied over a 6-week period, following a unilateral RET programme whereby one leg remained untrained throughout. The 1 repetition maximum (1-RM) for knee extension was determined for the dominant leg prior to the study beginning. On the first day of the study, subjects arrived at the laboratory following an overnight fast and body composition was assessed by dual-energy X-ray absorptiometry (DXA; Lunar Prodigy II, GE Medical Systems, Little Chalfont, Buckinghamshire, UK) before performing the first bout of RET. Thereafter, subjects returned to the laboratory 3 times per week to undertake supervised unilateral RET with 1-RM and maximum voluntary contraction (MVC) assessments of the trained leg ∼ every 10 d to ensure intensity progression. Thigh fat free mass (TFFM) was measured by DXA at 0, 3 and 6-weeks. Bilateral vastus lateralis (VL) biopsies were collected after an overnight fast at 0, 2, 4 and 6-weeks to assess MPS in discrete windows at 0–2 and 4–6 weeks with 200 ml of D_2_O consumed in 4 doses over 60 min (70 atom%; Sigma–Aldrich, Poole, UK) prior to biopsies at 0 and 4-weeks. Biopsies taken at 0 and 6-weeks were used to assess fibre cross sectional area (CSA) alongside myonuclei and satellite cell (SC) number. All biopsies were taken from the VL muscle 60–90 mins after a unilateral RE bout, under sterile conditions, using the conchotome biopsy technique [35] with 1% lidocaine (B. Braun Melsungen) as local anaesthetic. Muscle was rapidly dissected free of fat and connective tissue, washed in ice-cold PBS, blotted to remove excess water, frozen rapidly in liquid N_2,_ then stored at −80 °C until further analysis. Body water enrichment was monitored via saliva sample collection on RET-visits at least 30 min after their last meal or drink. These were collected in sterile plastic tubes and immediately cold centrifuged at 16,000 g to pellet any debris; they were then aliquoted into 2-ml glass vials and frozen at −20 °C until analysis. To measure levels of physical activity, subjects wore combined heart rate and activity monitors (Actiheart, CamMtech Ltd, Cambridge, UK) for 1 week. Actiheart data were checked for missing or extrapolated values; ≥80% of minute-by-minute physical activity data recording had to be available within any given 24 h period to be accepted, 5 subjects from each group had at least 2 or more days of data and were included in the analysis. Dietary intake was monitored by completion of 4-day diet diaries prior to and during the study and was analysed using Nutritics software (Nutritics, 2019).

**Fig. 1:**
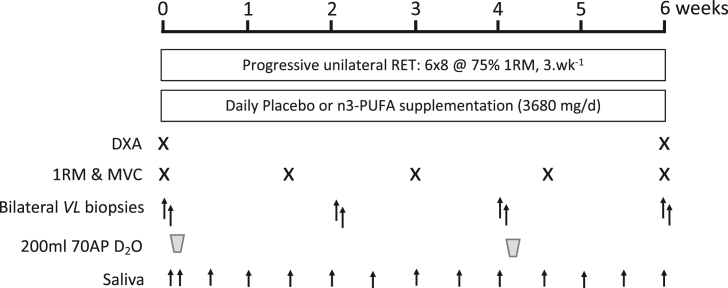
**Study protocol.** RET, resistance exercise training; 1RM, 1 repetition maximum; n-3 PUFA, n-3 polyunsaturated fatty acids; DXA, dual-energy x-ray absorptiometry; MVC, maximal voluntary contraction; *VL*, vastus lateralis muscle; AP, atom percent.

A detailed representative schematic of the study protocol is depicted in Fig. 1.

### 2.3 Treatment regime

All subjects were randomly assigned to either placebo (Cornoil) or n3-PUFA supplementation (3680 m g/d (1860 mg EPA; 1540 mg DHA) Minami Epacor). Both supplements were obtained in identical plain packaging, coded by the manufacturer, and all investigators remained blinded until study and analyses were completed. Subjects were first assigned to a lettered group, by a clinical measurement technician (AG, see acknowledgements), based on random allocation using a sealed envelope; then received the supplements weekly in daily pill boxes which were required to be returned to the investigators the following week as proof of compliance. Pill boxes demonstrated high supplementation compliance being 99% in placebo and n3-PUFA groups.

### 2.4 Muscle function assessments

Muscle function assessments were conducted using an isokinetic dynamometer (Isocom; Isokinetic Technologies, Eurokinetics) over a range of four knee joint angles (60°, 70°, 80° & 90°), with full extension corresponding to 0°. Leg extension 1-RM was assessed on a commercial grade plate-based leg extension machine (Technogym, Gambettola, Italy). Following explanation of the procedure, subjects performed a light warm up to avoid injury and ensure familiarity whilst avoiding fatigue. 1-RM was then achieved in as few repetitions as possible, within a maximum of 5 repetitions.

### 2.5 Muscle immunohistochemistry

**Fig. 2:**
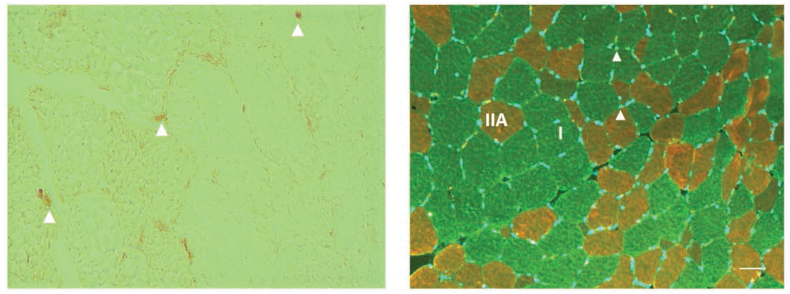
**Muscle immunohistochemistry.** Left, muscle cross section of human *m. vastus lateralis* antibody stained against Pax7 with satellite cells (arrowheads) visualized using diaminobenzidine (DAB) peroxidase kit. Right, immunofluorescent staining of human *m. vastus lateralis* for myosin heavy chain (MHC) type I (green), MHC type IIa (red) and laminin (green). Myonuclei are stained blue with DAPI (arrowheads) (scalebar = 50 μm). (For interpretation of the references to colour in this figure legend, the reader is referred to the Web version of this article.)

Muscle cross sections were cut at −20 °C with a cryostat microtome (Leica Biosystems, CM 1850). After 4h of air-drying at room temperature, sections were fixed with 2% paraformaldehyde and incubated in blocking buffer for 30 min. Satellite cells were stained using a mouse monoclonal abantibody against Pax7 (199,010 from Abcam). Sections were then incubated with a biotinylated secondary anti-mouse antibody followed by an incubation with Vectastain ABC reagent (Vector Laboratories, PK6100). Diaminobenzidine (DAB) peroxidase Substrate Kit (Vector Laboratories, SK-4105) was used for the visualization of the antibody binding. Immunofluorescence was subsequently used for the labelling of the basal lamina of muscle fibres and the identification of muscle fibre types. The slides were incubated for 60 min at 37 °C with the primary antibodies against laminin (D18), MHC IIa (SC-71) and MHC I (BA-F8) (Developmental Studies Hybridoma Bank). The following fluorescent labelled secondary antibodies were used: Alexa 488 for labelling of antibodies D18 and BA-F8 and Alexa 568 for labelling of the SC-71 antibody (LifeTechnologies). The slides were then mounted with Prolong gold antifade reagent with DAPI for staining of myonuclei in blue (Life Technologies). Satellite cells were stained brown and visualised using light microscopy. Type I muscle fibres were stained green, type IIA stained red, type IIX remained unstained (see Fig. 2).

### 2.6 Body water and protein bound alanine muscle fraction enrichment

Body water and muscle protein enrichment were measured as previously described [34]. In brief, pure fractions of water were extracted by heating saliva in a vial at 100 °C for 4 h, followed by cooling rapidly on ice and collection of condensate into a clean vial, ready for analysis. Deuterium enrichment was measured on a high-temperature conversion elemental analyzer connected to an isotope ratio mass spectrometer (TC/EA-IRMS; Thermo Scientific, Hemel Hempstead, UK). Myofibrillar proteins were extracted as previously described; briefly, the myofibrillar fraction was isolated by centrifugation from the sarcoplasmic protein, solubilized in 0.3M NaOH and separated from the insoluble collagen by centrifugation. The protein was then precipitated, acid hydrolysed, and the free amino acids purified on cation exchange resin and derivatised as their n-methoxycarbonyl methyl esters (MCME) [34]. Incorporation of deuterium into protein bound alanine was determined by gas chromatography-pyrolysis-isotope ratio mass spectrometry (GC-pyrolysis-IRMS, Delta V Advantage; Thermo Scientific, Hemel Hempstead, UK) alongside a standard curve of known dl-Alanine-2,3,3,3-d4 enrichment to validate measurement accuracy.

### 2.7 Calculation of muscle fractional synthetic rate

Myofibrillar MPS, representing the fractional protein synthetic rate, was calculated from the incorporation of deuterium-labelled alanine into protein, using the enrichment of body water (corrected for the mean number of deuterium moieties incorporated per alanine (3.7) and the dilution from the total number of hydrogens in the derivative (11) as the surrogate precursor labelling between subsequent biopsies. The equation used was:

FSR (%.d^−1^) = -Ln(1-((APE_ala_/APE_p_)/t))

where *APE*_*Ala*_ = deuterium enrichment of protein-bound alanine, *APE*_*P*_ = mean precursor enrichment over the time period, and *t* is the time between biopsies. The absolute synthetic rate was estimated using the equation:

ASR (g.d^−1^) = (FSR/100)∗ TFFM ∗ (12.4/100)

Assuming the myofibrillar protein content of skeletal muscle was 12.4% [36].

### 2.8 Immunoblotting

Immunoblotting was performed as previously described [37] using the sarcoplasmic fraction isolated from the MPS preparation described above. Sarcoplasmic protein concentrations were analysed using a NanoDrop ND1000 spectrophotometer (NanoDrop Technologies, Inc., Wilmington, DE-US) and sample concentrations adjusted to 1 μg/μl in 3x Laemmli buffer to ensure equivalent protein loading onto pre-cast 12% Bis-Tris Criterion XT gels (BioRad, Hemel Hempstead, UK) of 10 μg/lane. Samples were separated electrophoretically at 200 V for 1 h, followed by wet transfer of proteins to PVDF membrane at 100 V for 45 min and subsequent blocking in 2.5% non-fat milk in 1 Tris buffered saline/Tween 20 (TBST) for 1 h. Membranes were incubated in primary antibodies (1:2000 dilution in 2.5% BSA in TBS-T) overnight at 4 °C; p-mTOR Ser 2448 (#2972), p-4EBP1 Thr 37/46 (#2855), p-EEF2 Thr56 (#2331), Ubiquitin (1:1000 #3933) (New England Biolabs, Hertfordshire, UK) Calpain, (1:1000 ab3589) (Abcam, Cambridge, UK) MAFbx (1:1000 #AP2041) (ECM Biosciences, Versailles, KY, USA). Membranes were subsequently washed and incubated in HRP conjugated anti-rabbit secondary antibody (#7074, New England Biolabs, Hertfordshire, UK; 1:2000 in 2.5% BSA in TBST) at ambient temperature for 1 h, before being exposed to chemiluminescent HRP Substrate (Millipore Corporation, Billerica, MA-US) for 5 min and bands quantified by Chemidoc XRS (BioRad, Hertfordshire, UK). All signals were within the linear range of detection; loading was corrected to Coomassie [38].

### 2.9 Statistical analyses

Descriptive statistics were produced for all data sets to check for normal distribution using a Kolmogorov–Smirnov test. Where data was found to not be normally distributed, log transformation was performed. Data from all subjects (n = 8 per group) are presented as mean ± SEM or as a boxplots where the whiskers show the maximum and minimum, boxes represent the interquartile range, the cross indicates the mean and the horizontal line the median. All data sets were analysed by repeated measures two-way ANOVA with Bonferroni post-hoc testing using GraphPad Prism (La Jolla,CA) version 5. The α level of significance was set at P < 0.05.

## 3 Results

### 3.1 Changes in muscle function

**Fig. 3:**
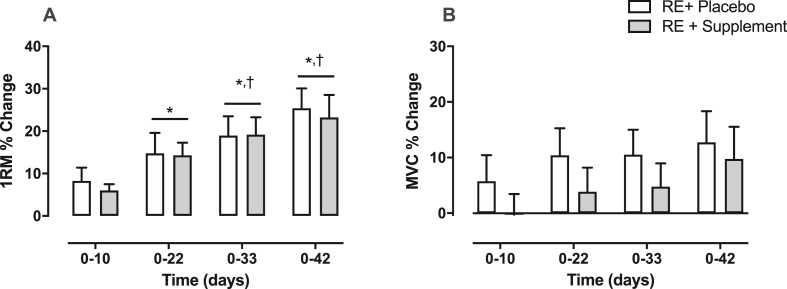
Time-course of strength (one repetition maximum (1RM) and maximum voluntary contraction (MVC) of the resistance exercise trained leg in both treatment (n-3 polyunsaturated fatty acids) and placebo groups. ∗ = P < 0.01 vs. baseline; † = P < 0.05 vs. day 10.

Over the 6-weeks of RET, the change in 1-RM increased progressively in the trained leg of both groups with a main effect of time (P < 0.001 (interaction: P = 0.9) (at 6-weeks PLA; +25 ± 5% (P < 0.01, MD = +6 kg, 95% CI = [3,9], d = 1.76) and n-3 PUFA; +23 ± 5% (P < 0.01, MD = +7 kg, 95% CI = [4,10], d = 1.40)) (Fig. 3A). However, there was no increase in MVC in the trained leg of either group at any angle (MVC 60° PLA; +13 ± 6% (P = 0.26, MD = +14 Nm, 95% CI = [−2, 31], d = 0.89) and n-3 PUFA; +10 ± 6% (P = 0.50, MD = +11 Nm, 95% CI = [−5, 27], d 0.63) (effect of time: P = 0.12, interaction: P = 0.9)) (Fig. 3B). There was no change in MVC in untrained legs throughout the study.

### 3.2 Activity levels, dietary intake and body composition

Monitoring of activity levels revealed no difference in activity counts between groups. There was no difference in dietary intake g/KgFFM (calories, protein, carbohydrates, fat) or n-3 PUFA consumption between groups at baseline (Table 2). During the study, n-3 PUFA intake was increased from baseline, and different from placebo with supplementation. We observed no other changes in dietary intake throughout the study. There was no effect of n-3 PUFA supplementation on body composition over the 6-weeks of supplementation at rest, with no changes in total mass, lean mass, bone mass or body fat percentage in either group (Table 3).

**Table 2:** Dietary intake before and during the study. Data as mean ± SEM. ∗ = P < 0.001 vs. Pre, § = P < 0.001 vs. Placebo.

		**Pre**	**Study**
**Calories** (g/KgFFM)	Placebo Supplement	39.8 ± 3.8 38.6 ± 3.1	41.0 ± 5.9 38.2 ± 1.6
**Carbohydrate** (g/KgFFM)	Placebo Supplement	4.2 ± 0.5 4.5 ± 0.3	4.1 ± 0.7 4.7 ± 0.3
**Protein** (g/KgFFM)	Placebo Supplement	1.9 ± 0.2 1.7 ± 0.2	1.9 ± 0.3 1.6 ± 0.1
**Fat** (g/KgFFM)	Placebo Supplement	1.5 ± 0.2 1.4 ± 0.2	1.6 ± 0.3 1.1 ± 0.1
**n3-PUFA** (g/KgFFM)	Placebo Supplement	0.029 ± 0.008 0.020 ± 0.008	0.024 ± 0.005 0.102 ± 0.003 ∗^,§^

**Table 3:** **Body composition.** Whole-body composition before and after 6-weeks unilateral resistance exercise training with fish oil supplementation (n-3 polyunsaturated fatty acids) or placebo.

	Baseline	6 Weeks
**Total Mass (Kg)**		
Placebo	64.3 ± 1.9	63.9 ± 1.9
n-3 PUFA	70.5 ± 2.6	69.7 ± 3.9
**Lean Mass (Kg)**		
Placebo	37.1 ± 1.6	36.7 ± 1.5
n-3 PUFA	39.4 ± 1.1	38.9 ± 1.1
**Body Fat (%)**		
Placebo	39.1 ± 1.6	39.4 ± 1.5
n-3 PUFA	40.8 ± 1.1	40.4 ± 0.9
**Bone Mass (Kg)**		
Placebo	20.1 ± 0.7	20.9 ± 0.7
n-3 PUFA	21.9 ± 0.6	22.2 ± 0.7

### 3.3 Thigh fat free mass and muscle fibre CSA

**Fig. 4:**
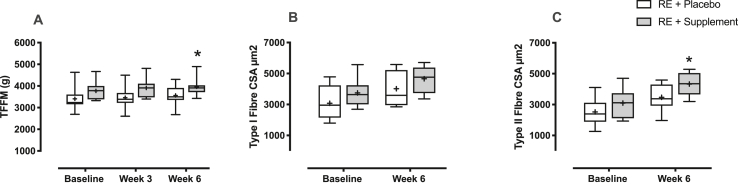
**Time course of changes in fat free mass and muscle fibre cross sectional area.** Changes in A) Thigh fat free mass (TFFM); B) Type I fibre cross sectional area (CSA); and C) Type II fibre CSA with resistance exercise (RE) training in both treatment (n-3 polyunsaturated fatty acids) and placebo groups. ∗ = P < 0.01 vs. baseline.

TFFM assessed by DXA was not significantly different between the groups at baseline (3406 ± 201 g PLA vs. 3774 ± 158 g n-3 PUFA). There was a main effect of time with RET (P < 0.01 (Interaction: P = 0.69)), yet only the n-3 PUFA group significantly increased TFFM in the trained leg (3961 ± 151 g (P < 0.05, MD = +187, 95% CI = [20, 355], d = 0.91)), with no change in PLA (3561 ± 170 g (P = 0.08, MD = +154, 95% CI = [−13, 322], d = 0.55)) (Fig. 4A). TFFM was unchanged in the control leg of both groups.

There were no differences in type I muscle fibre CSA at baseline (3078 ± 391 μm^2^ PLA vs. 3743 ± 323 μm^2^ n-3 PUFA). With RET there was a main effect of time (P < 0.05 (interaction: P = 0.97)), yet no increase after 6-weeks in either group (PLA; 4018 ± 403 μm^2^ (P = 0.14, MD = +939, 95% CI = [−256, +2135], d = 0.84)) and n-3 PUFA; 4659 ± 304 μm^2^ (P = 0.16, MD = +915, 95% CI = [−279, +2111], d = 0.61)) (Fig. 4B). There were no differences in type II muscle fibre CSA between the groups at baseline (2520 ± 316 μm^2^ PLA vs. 3097 ± 339 μm^2^ n-3 PUFA). However, there was a main effect of time (P < 0.01 (Interaction: P = 0.65)), with only n-3 PUFA showing an increase after 6-weeks of RET (4329 ± 264 μm^2^ (P < 0.05, MD = +1232, 95% CI = [205, 2258], d = 1.44), with no change in placebo (3467 ± 303 μm^2^ (P = 0.08, MD = +946, 95% CI = [−80, 1973], d = 1.08)) (Fig. 4C).

### 3.4 Myonuclei and satellite cell number

**Fig. 5:**
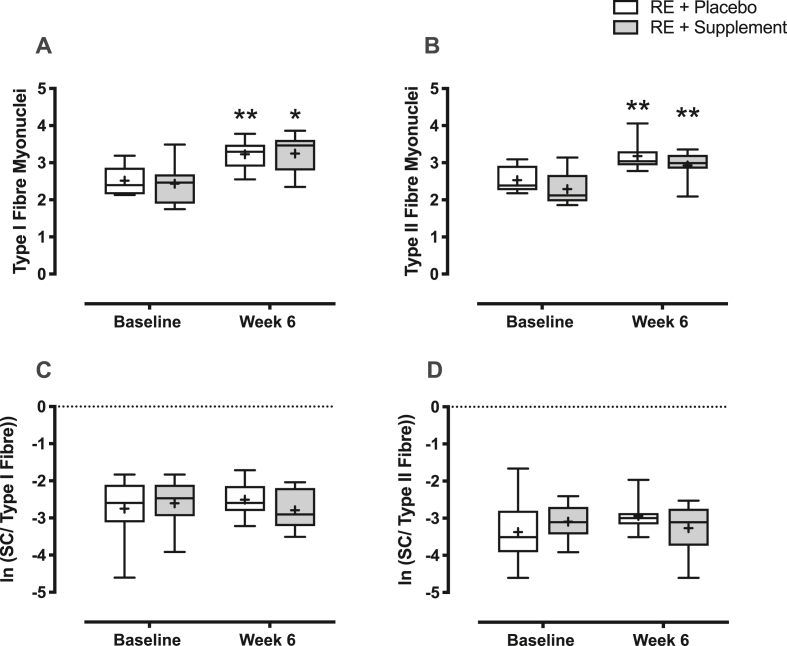
**Changes in myonuclei and satellite cell number.** Changes in A) Type I fibre myonuclei; B) Type II fibre myonuclei; C) Satellite cells (SC) per type I fibre; and D) Satellite cells per type II fibre with resistance exercise (RE) training in both treatment (n-3 polyunsaturated fatty acids) and placebo groups. ∗ = P < 0.05, ∗∗ = P < 0.01 vs. baseline.

There was no difference in type I fibre myonuclei number at baseline (2.5 ± 0.1 PLA vs. 2.4 ± 0.2 n-3 PUFA). There was a main effect of time with RET (P < 0.0001 (interaction: P = 0.77)), with both groups increasing after 6-weeks of RET (PLA; 3.2 ± 0.1 in PLA (P < 0.05, MD = +0.71, 95% CI = [0.16, 1.27], d = 1.78) and n-3 PUFA; 3.2 ± 0.2 (P < 0.01, MD = +0.81, 95% CI = [0.25, 1.37], d = 1.52) (Fig. 5A). Similarly, there was no difference in type II fibre myonuclei number at baseline (2.5 ± 0.1 PLA vs. 2.3 ± 0.2 n-3 PUFA). There was a main effect of time with RET (P < 0.0001 (interaction: P = 0.98)), with both groups increasing after 6-weeks of RET (PLA; 3.2 ± 0.1 (P < 0.01, MD = +0.65, 95% CI = [0.18, 1.12], d = 1.71) and n-3 PUFA; 2.9 ± 0.1 (P < 0.01, MD = +0.64, 95% CI = [0.17, 1.11], d = 1.53) (Fig. 5B). There was no change in the rest leg of either group.

There was no difference in type I fibre SC number at baseline (0.081 ± 0.017 [ln: −2.8 ± 0.3] in PLA and 0.086 ± 0.016 [ln: −2.6 ± 0.2] in n-3 PUFA with no change after 6-weeks of RET (time: P = 0.91, interaction: P = 0.36) (0.090 ± 0.016 [ln: −2.5 ± 0.2] in PLA (d = 0.18) and 0.070 ± 0.014 [ln: −2.8 ± 0.3] in n-3 PUFA (d = 0.038)) (Fig. 5C). Similarly, there was no difference in type II fibre SC number at baseline (0.051 ± 0.021 [ln: −3.4 ± 0.3] in PLA and 0.050 ± 0.008 n-3 PUFA [ln: −3.1 ± 0.2]) with no change after 6-weeks of RET (time: P=NS, interaction: P=NS) (0.059 ± 0.012 in PLA [ln: −2.9 ± 0.2] (d = 0.15) and 0.045 ± 0.008 in n-3 PUFA [ln: −3.3 ± 0.2] (d = 0.21) (Fig. 5D).

### 3.5 Muscle protein synthesis, absolute synthesis rate and muscle protein signalling

Over 0–2 weeks there was no difference in MPS in the untrained legs between PLA (1.15 ± 0.08%.d^−1^) and n-3 PUFA (1.25 ± 0.07%.d^−1^). With RET, there was a main effect of time (P < 0.001) (interaction: P = 0.3) with a significant increase in MPS in PLA (1.44 ± 0.09%.d^−1^ (P < 0.01, 95% CI [0.11, 0.48], d = 1.13) and approaching significance with n-3 PUFA (1.44 ± 0.072%.d^−1^ (P = 0.06, 95% CI [−0.005, 0.37], d = 1.20). At 4–6 weeks there was no difference in MPS in the untrained legs (PLA; 1.28 ± 0.10%.d^−1^ and n-3 PUFA; 1.22 ± 0.06%.d^−1^), with RET there was no effect of time (P = 0.07) or interaction: P = 0.86, with MPS remaining unchanged in both PLA (1.47 ± 0.09%.d^−1^ (95% CI [−0.14, 0.58], d = 0.57)) and n-3 PUFA (1.44 ± 0.11%.d^−1^ (95% CI [−0.18, 0.54], d = 0.42)).

**Fig. 6:**
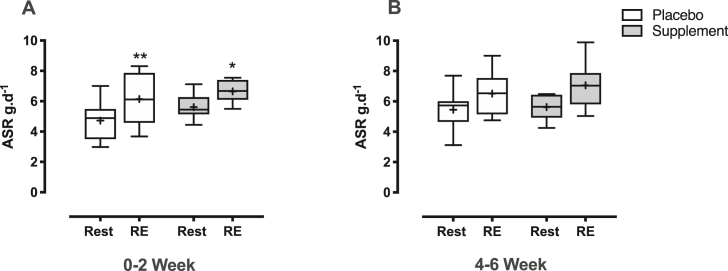
**Thigh absolute synthesis rates.** Thigh myofibrillar absolute synthetic rate in grams per day (ASR g.d^−1^) at rest and in response to resistance exercise (RE) training over 0–2 weeks (A) and 4–6 weeks (B) in both treatment (n-3 polyunsaturated fatty acids) and placebo groups. ∗ = P < 0.05, ∗∗ = P < 0.01 vs. baseline.

There was no difference in ASR between the untrained legs over the 0–2 week period (PLA; 4.7 ± 0.5 g d^−1^ and n-3 PUFA; 5.6 ± 0.3 g d^−1^). With RET, there was a main effect of time (P < 0.0001 (interaction P = 0.4)), with both groups showing a significant increase in ASR (PLA; 6.1 ± 0.6 g d^−1^ (P < 0.01, MD = +1.42, 95% CI = [0.61, 2.23], d = 1.19)) and n-3 PUFA; 6.7 ± 0.3 g d^−1^ (P < 0.05, MD = +1.03, 95% CI = [0.23, 1.84], d = 2.05))) (Fig. 6A). Similarly, ASR in the untrained legs was not different between groups over 4–6 weeks (PLA; 5.5 ± 0.5 g d^−1^ and n-3 PUFA; 5.6 ± 0.3 g d^−1^). With RET, there was a main effect of time (P < 0.05 (interaction (P = 0.7)), with both groups showing no change in ASR (6.5 ± 0.5 g d^−1^ (MD [95% CI] = +1.06 [−0.61, 2.73], d = 0.54))) but a trend towards an increase in n-3 PUFA (7.1 ± 0.5 g d^−1^ (P = 0.09, (MD [95% CI] = +1.44 [−0.23, 3.11], d = 0.80)) (Fig. 6B).

**Fig. 7:**
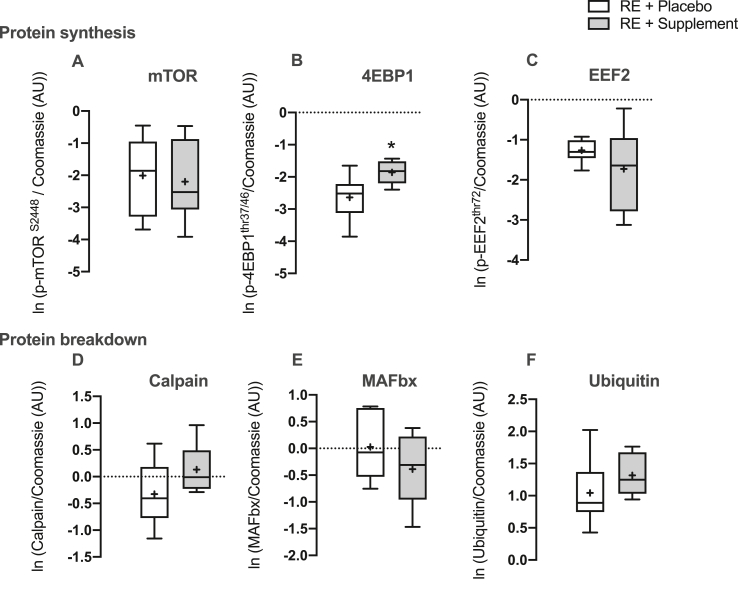
**Muscle protein signalling.** Muscle expression levels of markers of muscle protein synthesis: A) p-mTOR^s2448^ B) p-4EBP1^thr37/46^ C) p-EEF2^thr72^ and muscle protein breakdown: D) calpain E) MAFbx F) Ubiquitin 60–90 mins after the last bout of resistance exercise (RE) training in both treatment (n-3 polyunsaturated fatty acids) and placebo groups. ∗ = P < 0.05 vs. placebo.

The trend for an increase in ASR with RET in the n-3 PUFA group was supported by significantly greater activation of 4EBP1 (indicating increased cap-dependent mRNA translation) in n-3 PUFA versus placebo (Fig. 7B). There was no difference in expression of Calpain, MAFbx and Ubiquitin (markers of MPB), acutely after exercise at 6 weeks of RET between placebo and n-3 PUFA (Fig. 7D-F). There was no difference in activation of mTOR and EEF2 acutely after exercise at 6 weeks of RET between placebo and n-3 PUFA (Fig. 7 A,C).

## 4 Discussion

We and others have previously demonstrated that muscle strength and hypertrophy predominate in the early stages of a RET programme, and that these adaptations are blunted with ageing [2,32]. In the present study, 6-weeks of RET led to similar gains in muscle strength between placebo and n-3-PUFA supplementation in older women. However, n-3-PUFA specifically augmented muscle mass through modest increases in TFFM and type II fibre CSA. These findings support previous work highlighting the anabolic properties of n-3-PUFA in women [22,39] and suggest a role for n-3-PUFA in promoting anabolism by targeting type II fibre hypertrophy.

In the absence of RET, longer-term (4–6 month) n-3-PUFA supplementation alone has shown efficacy at increasing lean body mass [24], muscle thigh volume and strength [20]. However, after 6-weeks of n-3-PUFA supplementation in older women, using a within-subject unilateral rest/exercise model, we observed no change at the level of total body lean mass, nor thigh lean mass, VL fibre CSA or strength in the untrained leg. Therefore, it seems that n-3-PUFA supplementation alone is unlikely to have anabolic effects on muscle mass and function, at least over shorter supplementation periods i.e. up to 6 weeks.

Early epidemiological data indicated that fatty fish consumption had greater benefits on strength in women than men [28], yet the effect of n-3-PUFA supplementation on enhancing RET induced muscle hypertrophy has yielded equivocal results. Older women consuming a diet rich in n-3-PUFA alongside RET increased type II fibre CSA vs. a RET only group [39], whilst n-3-PUFA supplementation showed no added benefit on lean mass gains in men [27]. However, a mixed group of men and women showed no effect of n-3-PUFA in increasing thigh ACSA, measured using MRI [22]; yet since hypertrophy did not occur in any of these groups, it is possible that the frequency of the RET (2/wk^−1^) may have been insufficient.

The decline in muscle mass with ageing appears to be the result of both muscle fibre atrophy and fibre loss [40], yet whether one of these factors predominates remains unclear [41]. However, muscle fibre atrophy is frequently reported to be much greater in type II fibres than type I on older muscle [40,42]. This likely exacerbates declines in muscle strength observed in ageing, and combined with the fact that muscle atrophy is much greater in the lower body, will likely contribute substantially to greater functional decline and consequent loss of independence. The decline in type II fibre CSA has been highlighted and that RET interventions should primarily focus on developing effective strategies to minimize type II fibre CSA loss [43]. For the first time, we show that n-3-PUFA enhances early muscle mass gains in older women and that this is ostensibly driven by increases in type II fibre CSA, consistent with outcomes reported with long-term supplementation [39]. As such, these findings indicate that n-3-PUFA combined with RET may have clinical relevance as a strategy to mitigate muscle mass loss with ageing.

The addition of myonuclei has also been suggested to be important in skeletal muscle hypertrophy, yet whether they are essential remains a contentious issue [44,45]. Here we demonstrate that myonuclear addition occurred to the same extent in both groups, suggesting that skeletal muscle SC's remain responsive to contractile activity in older women. The diminished myofibre hypertrophy observed in the placebo group may be the result of impairments in translational efficiency/MPS, as we have previously reported in older men [2]. The lack of differences in strength gains we observed with RET between placebo and n-3-PUFA groups, in light of previously reported differential responses, may be due to the short-duration of both the adjuvant supplementation and RET. Muscle undergoes rapid architectural and neuromuscular adaptations in response to RET [46] and may result in early strength gains in the absence of hypertrophy [47]. As such, specific enhancements in strength in response to n-3-PUFA supplementation likely materialise after initial remodelling and require a longer study period to become apparent [21]. However, as muscle hypertrophy with prolonged RET is the primary driver of strength gains [48], early skeletal muscle hypertrophy with RET may have long term functional advantages, along with the many reported health benefits of increased muscle mass [49,50].

Chronic adaptations in skeletal muscle size are driven by acute and chronic changes in muscle protein turnover, yet the effects of n-3-PUFA supplementation on MPS and MPB are incompletely defined. Despite having no effect on fasted rates of myofibrillar MPS [[23], [24], [25], [26]], 8 weeks of n-3-PUFA supplementation enhanced MPS responses to feeding in both young and older men and women [25,26], suggesting that n-3-PUFA may bolster muscle responses to anabolic cues such as protein/amino acid nutrition and contractile activity. This led us to the approach of tracking the effects of supplementation upon integrated MPS, over 2-week ‘windows’ (i.e. between 0-2 and 4–6 wks). Initial MPS increases in response to RET can represent a heightened level of muscle protein turnover that may not be specific to muscle hypertrophy [51]. As such, exercise induced increases in MPS that do not result in increased muscle mass must be accompanied by an equivalent increases in MPB, perhaps as part of tissue remodelling [52]. Here, we observed no effect of n-3-PUFA supplementation on early (0–2 wk) RET MPS responses, with increased MPS in PLA representing increased muscle protein turnover in the absence of prevailing hypertrophy. Whilst MPS represents the fraction of new muscle being synthesised, ASR informs on the absolute amount of muscle myofibrillar protein content being synthesised. As RET induced increases in MPS diminish with continued RET [31,51], measures of ASR can provide additional insight into muscle hypertrophic adaptations. In response to RET, 4–6 wk MPS was not increased in either group, yet a trend for enhanced ASR with RET was observed, when supplementing with n-3-PUFA [25,26]. While few comparative studies exist, n-3-PUFA has previously showed a main effect of increased integrated myofibrillar protein synthesis (iMyoPS) with n-3-PUFA supplementation over a period of rest (3 d) then immobilisation (2 wk) and recovery (2 wk) [53]. However, these effects were primarily driven by an attenuation in immobilisation induced declines in MPS [53], indicating a potential role for n-3-PUFA in attenuating muscle mass loss during immobilisation [54].

Activation of anabolic signalling pathways that promote mRNA translation initiation and elongation are often taken as indicators of MPS activity. Previous work in growing steers showed that infusing n-3-PUFA rich menhaden oil for 35 days resulted in enhanced anabolic signalling via increases in fed state mTOR, P70S6K1 and AKT activation/phosphorylation [55]. This was confirmed in human muscle when 8 weeks of n-3-PUFA supplementation increased fed state mTOR and P70S6k1 signalling in both young and old individuals [25,26]. In support of this, 24h pre-treatment of C2C12 cells with EPA increased leucine stimulated MPS alongside P70S6K1 phosphorylation independently of mTOR or AKT [56]. As such, n-3-PUFA has shown efficacy for enhancing anabolic signalling pathways associated with translational efficiency, matching with increased protein synthesis. Herein we observed that n-3-PUFA supplementation enhanced the activation of 4EBP1 after a bout of RE in the trained leg, indicative of increased translation initiation, supporting the trend towards increased ASR and the myofibre CSA growth observed herein. In addition to translational efficiency, translational capacity (i.e. ribosome content) is important in regulating rates of protein synthesis and subsequent muscle adaptation [57]. Previously, we and others have demonstrated diminished ribosomal biogenesis to RET in older muscle that may be fundamental to the blunted muscle hypertrophy observed with age [2,58]. One way of increasing translational capacity is through SC mediated myonuclear addition and this has been purported to be essential for muscle hypertrophy. However, despite blunted hypertrophy in PLA, myonuclear addition was equivalent between groups, indicating n-3-PUFA may exert its effects primarily through enhancing translational efficiency rather than capacity.

In conclusion we have shown that n-3-PUFA supplementation enhances RET induced increases in muscle mass through type II fibre hypertrophy resulting in modest increases in TFFM. This adds to the previous literature on n-3-PUFA showing anabolic potency, particularly in women [22,39]. However, we also need to acknowledge some limitations within our study. Due to the purported benefits of n-3 PUFA in females, we did not study males, leaving the disparate gender responses to n-3 PUFA unclear. Further, other than the RET sessions and supplementation, subjects followed their habitual physical activity and dietary behaviours, which may increase inter subject variability. However, The activity levels of subjects within this study were similar to those we have previously reported [2] and subjects daily protein intake exceeded current recommendations [59]. The dietary recording revealed supplementation compliance was high, though we did not analyse plasma or muscle lipid n-3 PUFA ratios. Finally, although a strength of this study is the short term unilateral RE design, a longer-term, whole body RET intervention may provide additional insight into the longer-term efficacy if n-3 PUFA in mitigating sarcopenia and functional decline in ageing. Nevertheless, this data suggests n-3 PUFA supplementation may hold some benefit in combatting age related losses of muscle mass, however further, larger mechanistic time-course studies may be needed to establish the robustness these anabolic effects.

## Funding sources

The current work was funded by The Dunhill Medical Trust Grant R364/1112 (to KS, PJA, DJW, MVN & JPW), The Medical Research Council (grant numbers MR/R502364/1 and MR/P021220/1) as part of the MRC-Versus Arthritis Centre for Musculoskeletal Ageing Research awarded to the Universities of Nottingham and Birmingham, and the National Institute for Health Research (NIHR), Nottingham Biomedical Research Centre.

## Declaration of authorship

Study Design KS, PJA, DJW, JWW & MVN.

Conduct of Study UD, MB, AS, JQ, CB, HA, MF, BEP, JWW.

Laboratory Analyses & Functional Measures UD, MB, AS, JQ, MF, BEP, DJW, TJ, KF.

Data Collection and Analysis UD, MB, AS, JQ, MF, BEP, DJW, TJ, KF.

Interpretation and Writing Manuscript UD, MB, PJA, KS.

Read and approved Manuscript All authors.
